# Evolution and impact of molecular glue research: a bibliometric analysis from 2000 to 2023

**DOI:** 10.3389/fonc.2024.1401257

**Published:** 2024-05-16

**Authors:** Dian Li, Jingyi Cheng, Zhigen Yuan, Kexin Deng

**Affiliations:** ^1^ Department of Orthopaedics, Brain Hospital of Hunan Province (The Second People's Hospital of Hunan Province), Changsha, Hunan, China; ^2^ Department of Epidemiology, University of California, Los Angeles Fielding School of Public Health, Los Angeles, CA, United States; ^3^ Xiangya Stomatological Hospital and Xiangya School of Stomatology, Central South University, Changsha, Hunan, China; ^4^ Hunan Key Laboratory of Oral Health Research and Hunan Clinical Research Center of Oral Major Diseases and Oral Health and Academician Workstation for Oral-Maxilofacial and Regenerative Medicine, Central South University, Changsha, Hunan, China; ^5^ Department of Plastic and Reconstruction, The Third Xiangya Hospital of Central South University, Changsha, Hunan, China

**Keywords:** bibliometrics, molecular glues, targeted therapy, CiteSpace, visualization

## Abstract

**Background:**

Molecular glues, which reshape E3 ligase receptors to promote targeted protein degradation, are emerging as a promising therapeutic strategy, particularly in oncology, driven by rapidly advancing insights into their mechanisms and structural properties.

**Objective:**

This study aims to offer an insightful depiction and visualization of the knowledge structure, prevalent themes, and emerging trends within the domain since the year 2000, employing bibliometric analysis to achieve this goal.

**Methods:**

To conduct this research, a comprehensive collection of literature on molecular glues was sourced from the Web of Science database. Subsequently, the data underwent analysis utilizing CiteSpace and VOSviewer tools, enabling the identification of pivotal countries, institutions, authors, and journals, as well as the delineation of subject hotspots, trends, and the forefront of research in this evolving field.

**Result:**

Since 2000, 388 papers on molecular glues have been published, with a notable increase to an annual average of 43 articles post-2018. This research, contributed by 506 authors across 329 institutions, highlights the United States and China as leading nations in output, with 122 and 104 articles respectively. Takuzo Aida, Luc Brunsveld, and Christian Ottmann are identified as key authors. Nature emerges as the foremost publication venue, while the Chinese Academy of Sciences is the top contributing institution, underscoring the global engagement and interdisciplinary nature of molecular glue research. This study identified 19 distinct research clusters within the molecular glues domain.

**Conclusion:**

We reveal the current status, hotspots, and trends of molecular glue research since 2000, offering insights and novel scholarly perspectives on the field’s prevailing limitations.

## 1 Introduction

Protein homeostasis, also termed proteostasis, is a highly intricate and interconnected cellular process maintaining concentration, conformation, and subcellular localization of proteins, which encompasses a series of regulatory processes, including protein synthesis, folding, transport, and degradation (1). It acts as an essential aspect of cell function and viability, the disorder of which has been associated with a wide range of human pathologies, especially age-related diseases such as neurodegenerative diseases and cancers (2, 3). The ubiquitin-proteasome system (UPS) is a master protein homeostasis regulator, eliminating damaged, unfolded, and misfolded proteins through a ubiquitin-dependent E1-E2-E3 enzymatic cascade (4). In this cascade, ubiquitin ligase E3 is the crucial component, catalyzing the transfer of the ubiquitin from E2 to specific substrates.

Currently, it is constantly exploring how to degrade the proteins of interest through the inherent protein degradation mechanism, and targeted protein degradation (TPD) as a pioneering strategy has generated tremendous excitement in chemical biology and drug discovery. TPD exhibits some prominent advantages compared to traditional pharmacological target protein inhibition (5–7): (1) increased efficiency at very small doses, as a single degrader can catalytically obliterate numerous copies of a pathogenic protein; (2) higher sensitivity to drug-resistant targets, as degraders invalidate all the functions of pathogenic proteins once the active site is blocked; (3) availability for pathogenic protein targets that were once considered to be “undruggable.”Most TPD strategies depend on the UPS. Among them, proteolysis-targeting chimeras (PROTAC) (8) and molecular glue (9) are two representative techniques developing rapidly.

Molecular glues refer to drugs or small molecules that regulate protein functions and metabolism by enhancing the interactions between proteins. Unlike traditional enzyme inhibitors or receptor agonists, these molecules primarily function by promoting the binding between proteins and can potentially alter their conformation or functionality, thereby impacting cellular signaling and metabolic processes. In 2007, Professor Ning Zheng and his team from the University of Washington published a study in Nature that enriched our understanding of molecular glues (10). This research revealed their mechanism of action within the context of E3 ubiquitin ligases, showing that these molecules can remodel the surface of E3 ligase receptors to foster new protein-protein interactions, subsequently mediating protein degradation induced by proximity. Molecular glues directly enhance the assembly by squeezing the E3 ligase and the target protein interfaces instead of a flexible linker like PROTAC (11, 12). Molecular glue presents better pharmacological properties compared to PROTAC, as a smaller molecular weight contributes to higher membrane permeability and better cellular uptake (13). Lenalidomide and its analogs, such as thalidomide and pomalidomide, also known as immunomodulatory imide drugs (IMiDs), are identified as a kind of molecular glue that reprograms E3 ligase cereblon to degrade zinc finger transcription factors (e.g., IKZF1/3, CK1α) (14, 15). Antitumor aryl-sulfonamides were found to be molecular glues that modify DCAF15, another E3 ligase, to degrade specific RNA-binding proteins (16). These classical molecular glue degraders are identified serendipitously, and there is still a lack of rational discovery and design strategies for molecular glues. However, an advanced understanding of the precise mechanism, structural biology, and medicinal chemistry features of molecular glues are emerging rapidly, and it is conceivable that molecular glues will attract more attention as a promising new therapeutic strategy from the fields of biomedicine and the pharmaceutical industry.

Bibliometrics emerged in the early twentieth century and was established as an independent discipline by Pritchard in 1969, gaining widespread application in the analysis of scientific literature (17, 18). Defined by Mayr and Scharnhorst (19) as a quantitative approach to investigating, examining, and analyzing research outputs within a specific domain, bibliometric analysis aims to extract comprehensive information such as authors, keywords, references, institutions, countries, etc. Such data facilitates further analyses concerning properties and performance, thus evaluating the progression within particular fields (20). With the advancement of modern imaging and visual technologies, literature analysis has been enhanced to offer more intuitive insights. Presently, the foundation and focus of bibliometric visualization analysis lie in co-citation analysis. The principle that one or more subsequent articles cite two articles together establishes a co-citation relationship. It extends analogously to authors, journals, keywords, countries, institutions, etc., allowing for the exploration and visualization of their interconnections. This approach has proven useful for identifying shared research directions among journals, common topics among authors, and more, thereby rendering co-citation visual measurement a reliable and comprehensive method for data interpretation in bibliometrics, as advocated by Ma and Xi (21).

This paper aims to furnish a detailed and systematic examination of scientometrics within the molecular glues domain, focusing on aspects such as collaborative networks and co-emergence among states, institutions, authors, etc. It also meticulously reviews the temporal and spatial shifts in research priorities and frontier trends within the field. Utilizing CiteSpace, a citation analysis tool that draws on data from the Web of Science Core Collection (WoSCC), this study supports scientific inquiry into skin and probiotics, addressing the knowledge gap in bibliometric reviews of the subject (22). Specifically, this research (1) delineates the scholarly discourse on molecular glues since the turn of the millennium, contextualized within global collaboration at various structural levels, including authors, institutions, and countries; (2) identifies and discusses the predominant research themes and their distinguishing features; and (3) synthesizes the overall developmental trajectory and distinct characteristics of the field, analyzing research avenues with significant potential based on trend analysis.

## 2 Methods and data

### 2.1 Research methods

Bibliometrics, a specialized branch of informatics, presents a methodologically sound and objective approach to analyzing and reviewing research outputs within specific fields. Through the structured examination of extensive datasets, it effectively maps out the research landscape, organizes research themes into clusters, tracks shifts in research directions, and pinpoints the most influential authors, institutions, and countries (22). Enhanced by modern digital and visual analytics, systematic scientometric methodologies significantly improve the efficiency, accessibility, and reliability of bibliometric research across academic disciplines (23).

In this realm, bibliometrics excels at unveiling and integrating the hidden knowledge structures of academic work into accessible visual representations, thereby facilitating deeper analytical exploration. CiteSpace stands out with its comprehensive, time-sensitive, and dynamic citation visualization features, allowing for an intuitive understanding of complex research domains. Its analytical arsenal includes co-citation analysis, keyword co-occurrence analysis, burst detection, and cluster analysis, each serving distinct purposes (24). Co-citation analysis explores the relationships between studies through their mutual citations, where a higher frequency of co-citations suggests greater similarity and stronger linkage (25). Keyword co-occurrence analysis quantifies the frequency of keyword appearances, assessing their relational proximity based on co-occurrence (24). Burst detection uncovers trends in the use of specific keywords, signaling shifts in research focus (22), while cluster analysis organizes objects based on similarity, enabling detailed analysis of formed clusters (25). Centrality, a critical measure, assesses keyword significance, with nodes of centrality above 0.1 deemed central and hence more pivotal in the research landscape (26). This could position an article as a key intermediary between two others or highlight a keyword as a crucial link among various studies, underlining its central role in the academic discourse. By bridging cognitive gaps and emphasizing key findings and emerging trends, CiteSpace facilitates comprehensive navigation through the research field (27). Thus, this study employs a bibliometric approach via CiteSpace to explore connections with relevant literature comprehensively, undertaking critical reviews to probe deeply into significant research areas and extract essential insights on the topic.

### 2.2 Data resource

**Figure 1:**
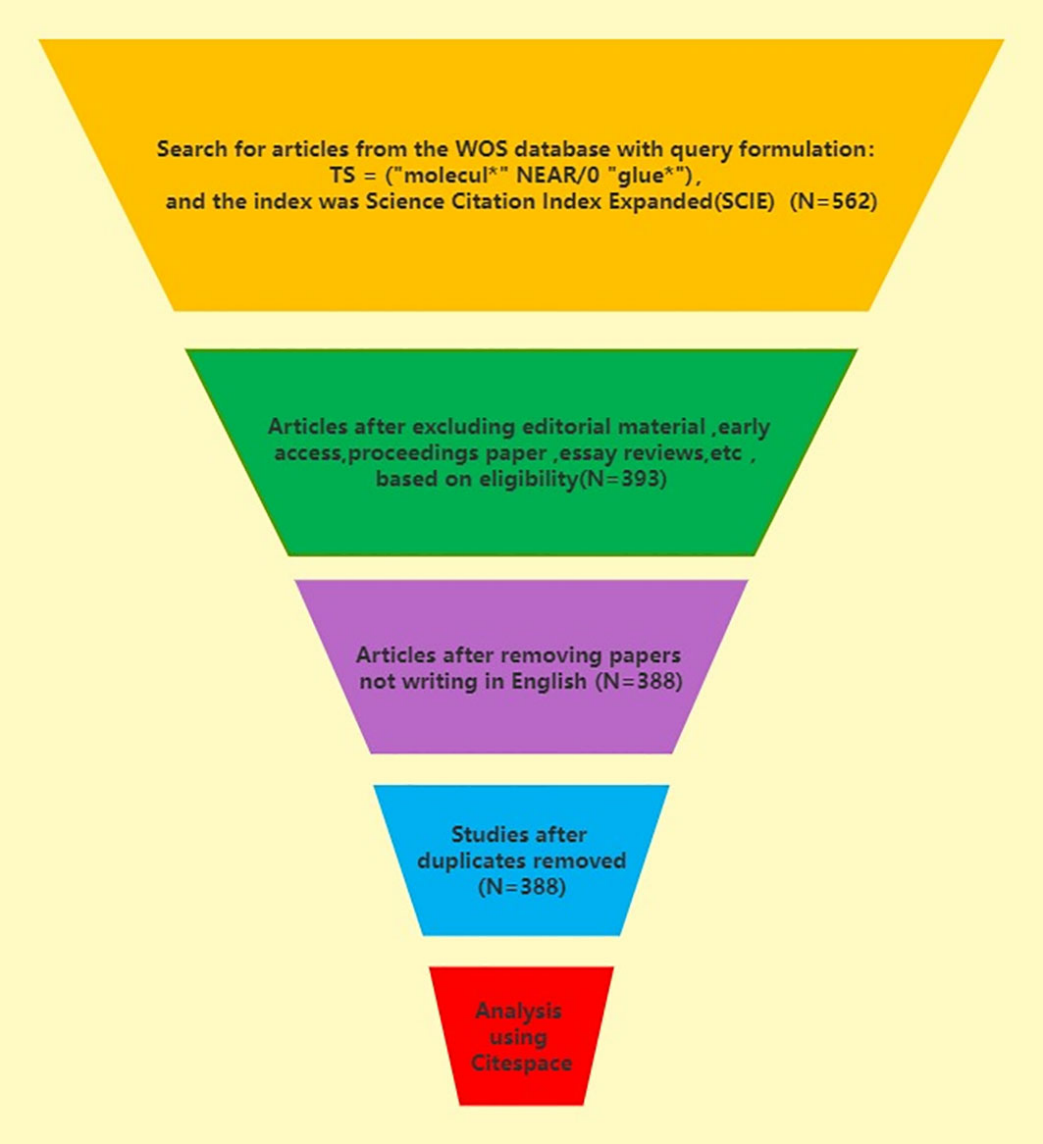
The search strategy used for the present bibliometric analysis.

In this paper, Web of Science Core Collection (WoSCC) was selected and the index was Science Citation Index Expanded(SCIE). The retrieval formula in this paper was as follows: TS = (“molecul*” NEAR/0 “glue*”). The time span was from January 1st, 2000 to December 31st, 2023. A total of 562 publications (393 articles, 118 reviews, 19 conference abstracts and 32 others) were searched on January 1st, 2024, and 388 articles were selected that are articles and writing in English after removing duplicates. This paper made a bibliometric analysis of the 388 articles. The literature retrieval and screening process is recorded in **Figure 1** and **Table 1**.

**Table 1:** The search strategy used for the present bibliometric analysis.

Category	Specific standard requirements
Research database	Web of science core collection
Citation indexes	SCIE
Searching period	2000-01-01 to 2023-12-31
language	“English”
Document types	“Articles”
Data extraction	Export with full records and cited references in plain text format
Query formulation	TS = (“molecul*” NEAR/0 “glue*”)
Sample size	388

## 3 Results

### 3.1 Analysis of publishing trend

**Figure 2:**
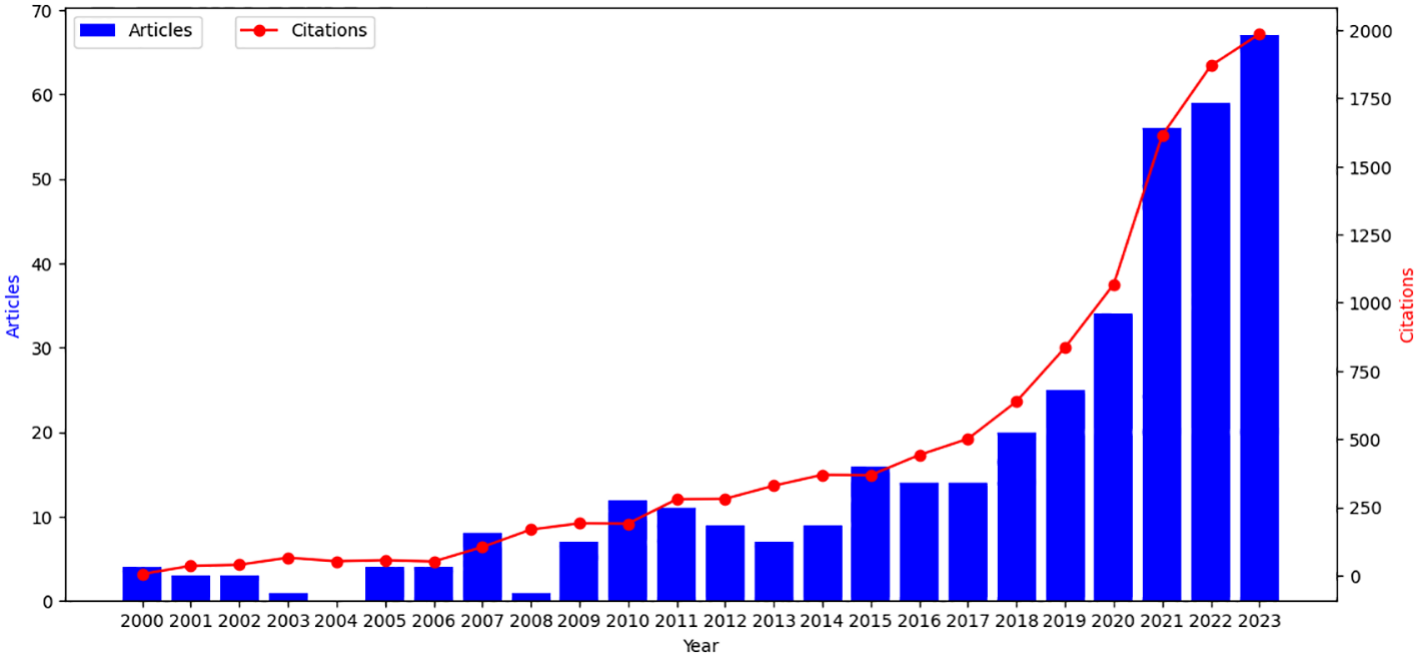
Time evolution of the total number of publications and citations in the WOS database, The ordinate represents the number of published papers, while the abscissa represents the years. The overall trend of the line chart shows an upward trend.

The trend of literature publication is an important index to measure the research development in a certain field. Therefore, by drawing the quantity-time curve of literature, we could effectively evaluate the research status in this field and further predict the development trend. **Figure 2** shows the annual distribution of articles related to molecular glue on Web of Science since the 21st century. It was calculated that the average number of articles was only 16 per year since the 21st century. On the whole, there has been significant growth and progress in the study of molecular glue. In the past six years, the number of articles and citations on molecular glue has maintained a steady growth rate. Before 2018, the number of articles was relatively small, only about 7 articles a year. It began to increase rapidly after 2018, with an average of about 43 articles. In the last three years, it has reached 60 articles per year. The total citation number has also increased from less than 5 to 1986. Although the number of studies has not reached a high level, the research on molecular glue is developing rapidly. At the same time, according to the research trend, there will still be a lot of space in this field in the next few years, and the research will continue to grow.

### 3.2 National, institutional, author and journal analysis

#### 3.2.1 National analysis

**Figure 3:**
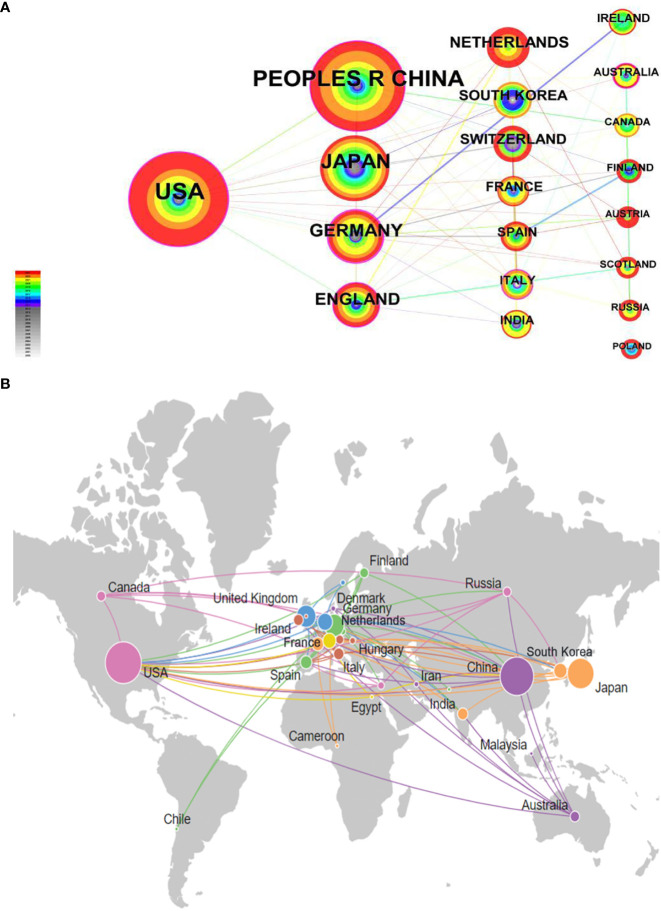
Analysis of publication among countries. **(A)** Cooperation network of countries. The “Time Slicing” was set to “2000-2023” with a “Years Per Slice” of “1”. In the visualization, the purple outer ring represents countries with an intermediary centrality greater than 0.1. Intermediary centrality is a measure of the importance of the research object in the collaborative network, indicating the strength of its bridging role in connecting multiple other countries. The size of the nodes corresponds to the volume of publications, with countries that have a higher number of publications being represented by larger nodes. **(B)** Analysis of geographical distribution of countries. This image in Citespace was created by connected to Google Maps. The size of the nodes in the visualization corresponds to the number of publications, with larger nodes indicating more publications from a country. To improve the visual appearance, connections between countries with fewer collaborations were left out.

Through the quantitative analysis of countries, we could not only identify the core countries in the research field of molecular glue but also reflect the academic communication and cooperation between countries in this field. In this study, the “Country” was selected as the analysis object in CiteSpace, the time “Time Slicing” was “2000-2023”, the “Years Per Slice” was “1”, and the threshold was top “20”. Finally, a national analysis map with 38 network nodes and 108 connections with a density of 0.1536 was obtained, as shown in **Figure 3A**. The thickness of the purple ring indicates the degree of its intermediate centrality, reflecting the importance of its position in the network.

The top 20 countries are listed in **Table 2**. The frequency represents the count of publications in the country, and the centrality represents the importance of the country in this field. It can be seen in **Figure 3A** that the more international connections there are, the higher the centrality and the greater the authority the country has.

**Table 2:** The centrality and count of literature in countries.

Country	Year	Frequency	Centrality
USA	2000	122	0.24
PEOPLES R CHINA	2010	104	0.17
JAPAN	2006	66	0.01
GERMANY	2005	36	0.43
ENGLAND	2002	29	0.14
NETHERLANDS	2010	21	0.06
SWITZERLAND	2007	16	0.03
SOUTH KOREA	2001	16	0.01
FRANCE	2005	13	0.05
SPAIN	2005	12	0.05
ITALY	2000	10	0.11
INDIA	2011	10	0.02
IRELAND	2015	9	0.03
AUSTRALIA	2012	8	0.12
CANADA	2002	7	0.01
FINLAND	2011	7	0.01
RUSSIA	2018	6	0.03
SCOTLAND	2010	6	0.03
AUSTRIA	2007	6	0.01
POLAND	2010	5	0

’Year’ denotes the initial publication year, ‘Frequency’ indicates the number of publications, and ‘Centrality’ refer to intermediary centrality.

As can be seen from the frequency of **Table 2**, the USA has been the country with the highest number of articles in this field, having 122 related research, which was much higher than that of other countries, indicating that the USA has paid great attention to the research field of molecular glue and been in a leading state. Meanwhile, GERMANY has also been the country with the highest intermediary centrality, about 0.43, which indicates that GERMANY’s transnational cooperation in this area was most common and has been in a leading position. The frequency of PEOPLES R CHINA ranked second; 104 related studies were published, and JAPAN ranked third with a frequency of 66. From the perspective of centrality, Japanese scholars have tended to research independently. At the same time, the intermediary centrality of the USA, CHINA, Germany, England, ITALY, and AUSTRALIA also broke through 0.1, indicating that research in these countries has often been done by transnational cooperation. **Figure 3B** shows that most of the studies have concentrated in developed countries except China. Additionally, it can be seen from **Table 2** that the countries whose intermediary centrality is more significant than 0.1 were almost in European and American developed countries. So, the developed countries playing an absolute leading role in the field have paid more attention to research in the field and added weight to international cooperation. From the starting time, we could know that the research of the USA and ITALY appeared earliest, and their centrality was also higher, which showed that research in these two countries had played the foundation role.

#### 3.2.2 Institutional analysis

**Figure 4:**
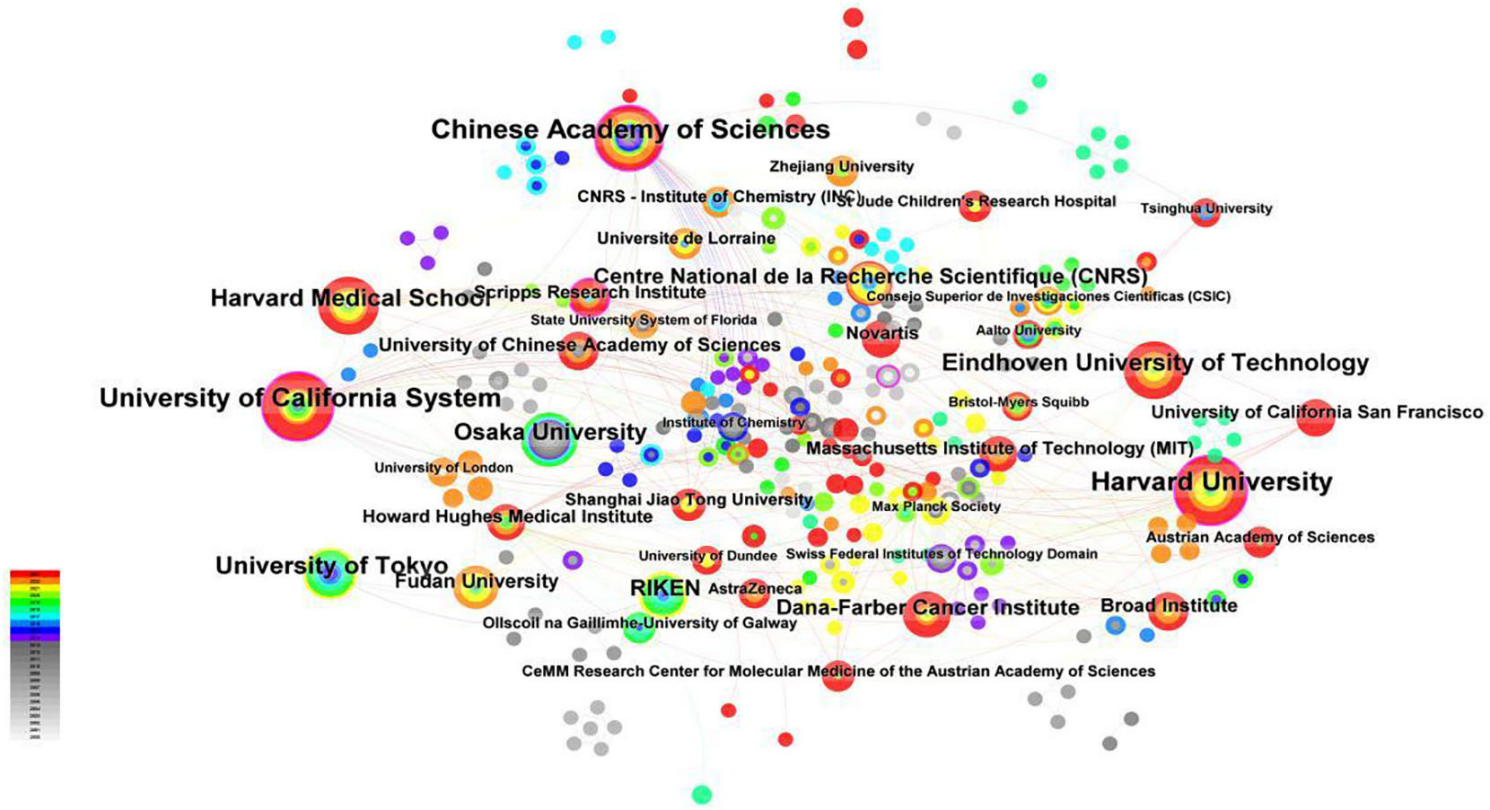
Interinstitutional cooperation network. Using ”Institution” in CiteSpace as the analysis object. The “Time Slicing” was set to “2000-2023” with a “Years Per Slice” of “1”. In the visualization, the purple outer ring represents institutions with an intermediary centrality greater than 0. The size of the nodes corresponds to the frequency of article publications by each institution. To enhance the visual appeal of the image, the lines between institutions with relatively few collaborative instances have been omitted.

Using “Institution” in CiteSpace as the analysis object, an institutional analysis map with a density of 0.0111 with 329 network nodes and 597 connections was obtained. As shown in **Figure 4**, the nodes in the map are relatively dense, and most of the data are connected, with a total connection of 479, which indicates that institutional cooperation in the field of molecular glue has been relatively frequent, and most research institutions have had cooperation. Some of these institutional cooperations had obvious regional characteristics, which showed that inter-institution research needs to be maintained to promote academic exchanges in the field of molecular glue.

This paper also collated the top 10 institutions in the field of molecular glue. As can be seen from **Table 3**, *the Chinese Academy of Sciences, Harvard University*, and *the University of California System* were the top 3 institutions, with a volume of 24,23, and 23, leading other institutions. However, the centrality of *the University of Tokyo* was 0, which showed that the University of Tokyo had a relatively higher frequency of publication but less cooperation with other institutions. It can also be seen that the publication times of *Harvard University* and its affiliated medical school are earlier than others, indicating that the organizations have played a leading role in the research of molecular glue.

**Table 3:** The top 10 institutions of publication.

Institution	Year	Freq	Centrality
Chinese Academy of Sciences	2010	24	0.19
Harvard University	2000	23	0.15
University of California System	2002	23	0.13
University of Tokyo	2009	18	0
Harvard Medical School	2000	16	0.08
Eindhoven University of Technology	2020	16	0.01
Osaka University	2006	15	0.04
RIKEN	2015	13	0
Centre National de la Recherche Scientifique (CNRS)	2005	12	0.09
Dana-Farber Cancer Institute	2020	12	0

’Year’ denotes the initial publication year, ‘Frequency’ indicates the number of publications, and ‘Centrality’ refer to intermediary centrality.

Although the *Eindhoven University of Technology* paid attention to the research field of molecular glue relatively later, the number of articles published in it has also been relatively higher. The three institutions with the highest intermediary centrality have been *the Chinese Academy of Sciences, Harvard University*, and *the University of California System*, respectively. At the same time, they are the three institutions with the highest number of publications. It could be seen that stronger institutional cooperation is conducive to the generation of scientific results, especially the molecular glue, which is the intersection of materials and biomedicine.

#### 3.2.3 Author cooperation analysis

**Figure 5:**
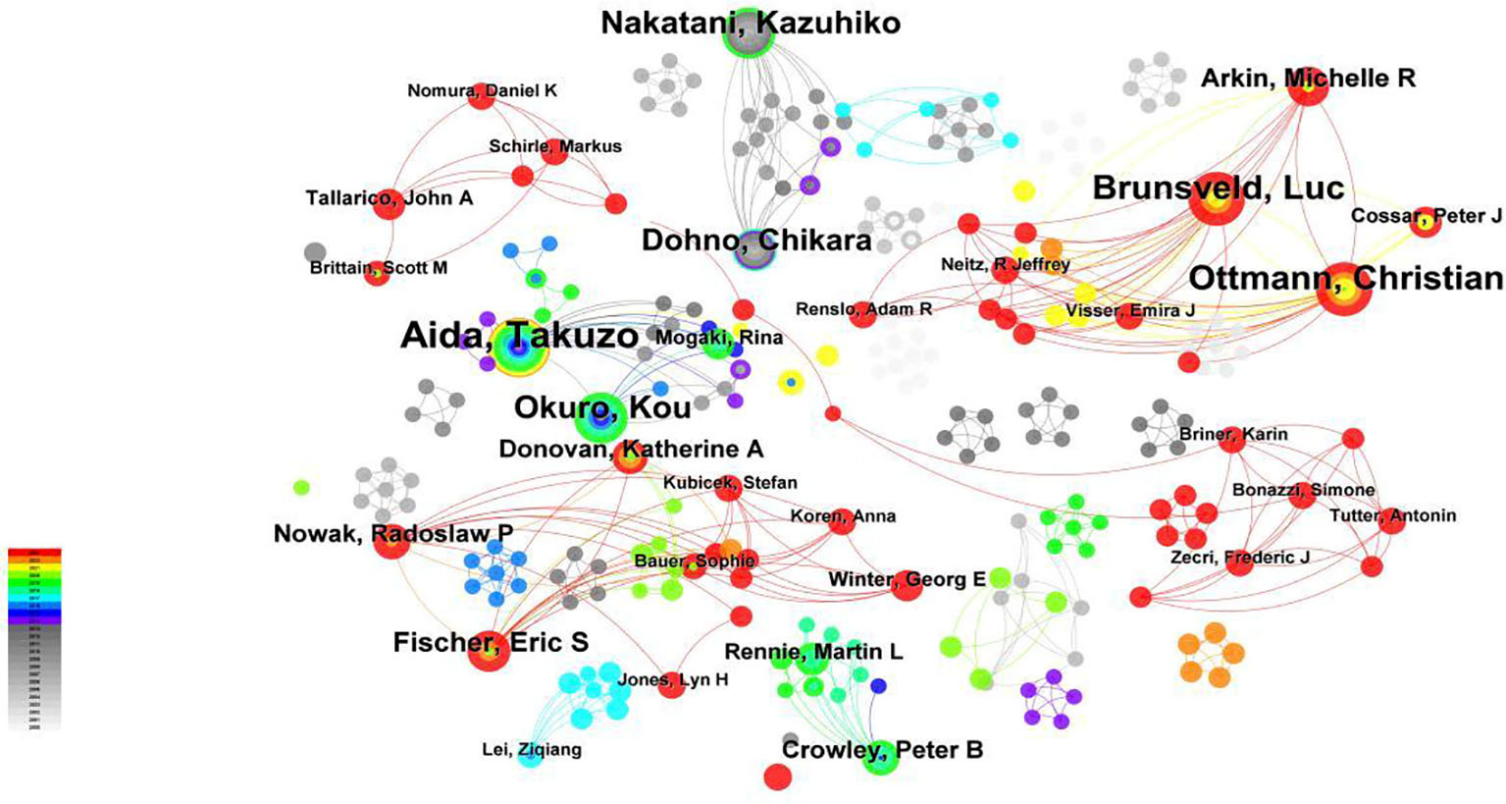
Author publication volume and collaboration analysis. Using ‘Author’ as the object of analysis in CiteSpace, we generated an analysis map of author cooperation and publications. The size of the nodes corresponds to the frequency of article publications by each author. The lines between institutions with relatively few collaborative instances have been omitted.

Using ‘Author’ in CiteSpace as the analysis object, an author cooperation analysis map with 506 network nodes, 886 connections, and a density of 0.0069 was obtained (**Figure 5**). A total of 388 articles were published by 506 authors, with 886 collaborations. As can be seen from **Figure 5**, the three most prominent nodes of authors were Aida, Takuzo; Brunsveld, Luc; Ottmann, Christian, with publication numbers of 20,14 and 14, respectively, indicating that they were the three most influential authors in the field.Brunsveld, Luc and Ottmann, Christian have been very productive. In addition, they are two scholars who have collaborated closely with each other, and their research has also been at the forefront. There has been no connection between the two of them, and Aida, Takuzo. Aida Takuzo”s study was older. Besides, the time of cooperation and publications of a large number of authors have been relatively close to the present, which shows that the biomedical applications of molecular glue have been proliferating. The scholars have been active and very communicative in recent years.

#### 3.2.4 Journal co-citation analysis

**Figure 6:**
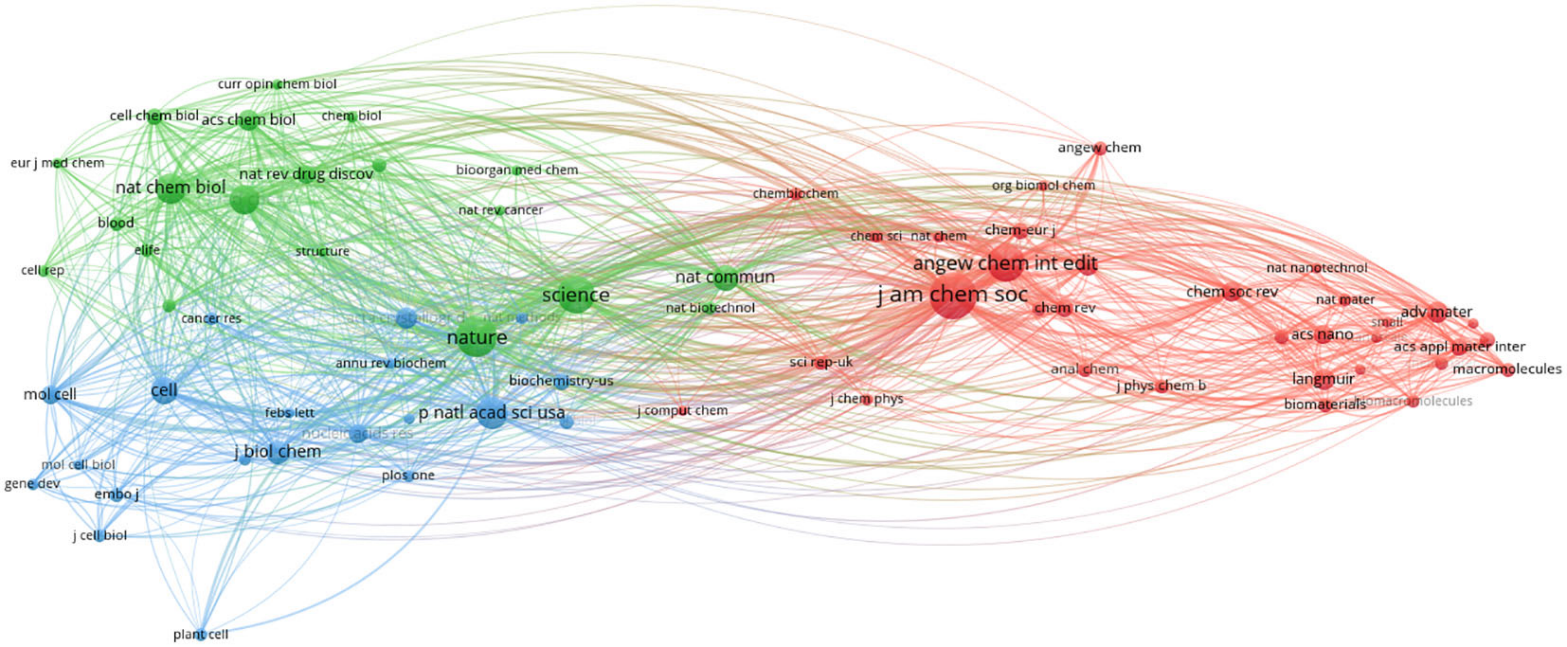
Analysis diagram of journal co-citation network. Each node represents a journal, with larger nodes indicating a greater number of published articles. Journals of the same color are indicative of closely related topics in their published articles, and the lines connecting the nodes represent their co-appearance in citations within the same articles.

Using VOSviwer’s to generate a journal co-citation network graph and publications and citations table (**Figure 6**, **Table 4**), we found three core journals that are the most authoritative in the field. By observing the node size in **Figure 6** and **Table 4**, we could intuitively see the journals that publish the most articles in this field. **Table 4** lists the top 10 journals in the field of molecular glue and their citation value. From the comparison of journal publications, citations, or impact factors, the core journals of molecular glue have been Nature, Journal of the American Chemical Society, and Angewandte Chemie-International Edition. Nature is the leading research journal in the fields of biology and chemistry, encompassing the most cutting-edge scientific research. Journal of the American Chemical Society and Angewandte Chemie-International Edition have been the top journals in the field of chemistry and contributed greatly to research in the field of molecular gums.

**Table 4:** Journals with the top10 citations.

Rank	Journals	Publications	Number of citations
1	Nature	5	1532
2	Journal of the American Chemical Society	26	761
3	Angewandte Chemie-International Edition	16	468
4	Langmuir	8	366
5	Nature Chemical Biology	7	364
6	Proceedings of the National Academy of Sciences of the United States of America	5	342
7	Nature Communications	12	299
8	Journal of Medicinal Chemistry	15	294
9	ACS Applied Materials & Interfaces	5	173
10	Soft Matter	5	169

”Publications” represents the number of articles published in the journal “Number of citations” refers to the total number of citations received by the relevant articles published in the journal.

### 3.3 Keywords cluster and co-citation analysis, frontier and trend analysis

#### 3.3.1 Keywords cluster and co-citation analysis

**Figure 7:**
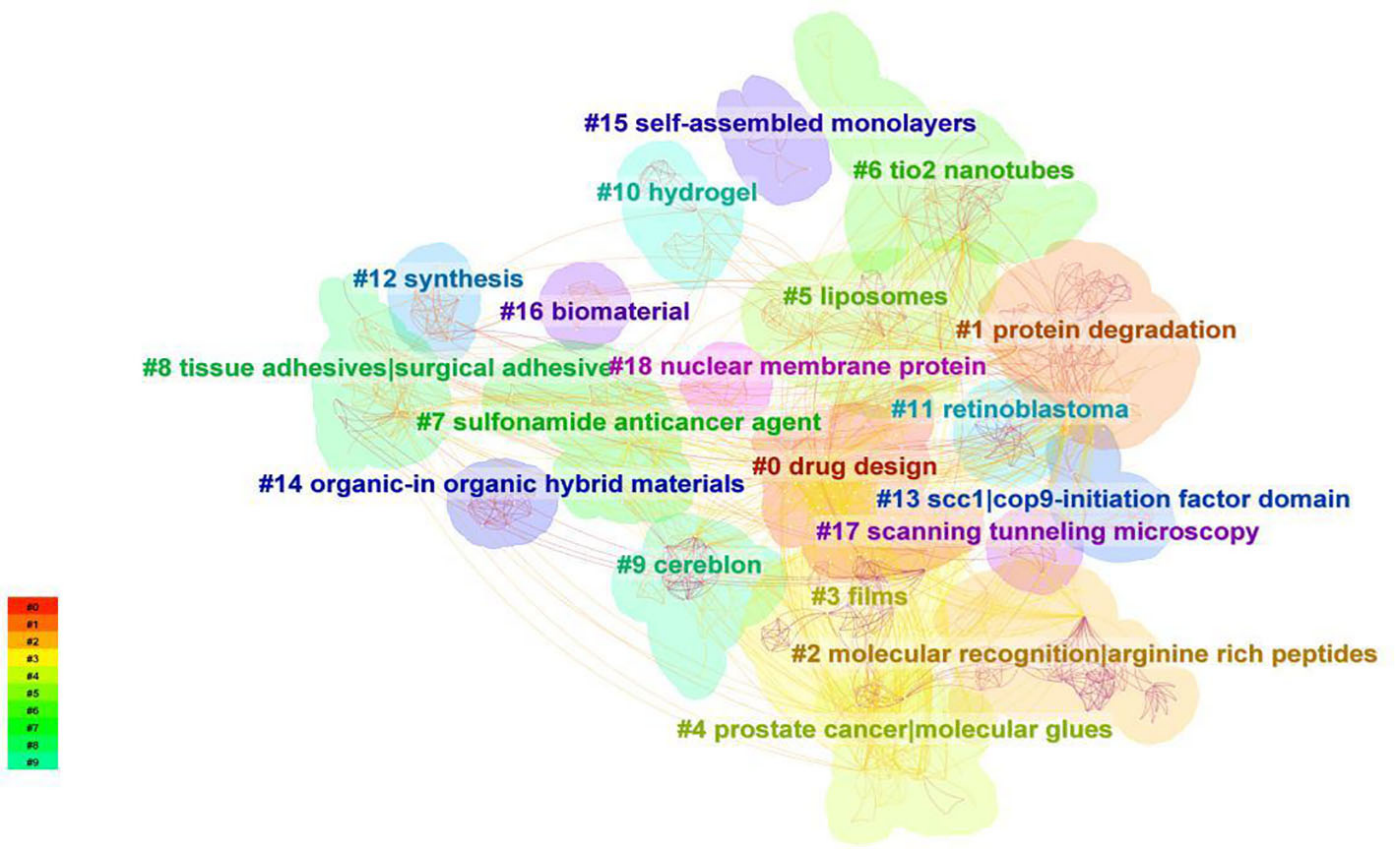
Analysis of keywords clustering. ‘Cluster’ was used as the object of analysis in CiteSpace, and the pathfinder algorithm was used to cut the connection lines to ensure the classification rationality of clusters. A total of 19 keyword clusters have been identified, with each cluster assigned a different color based on the time in the bottom left corner. The cluster names are derived from representative keywords obtained using the LLR algorithm.

In this paper, we used Citespace to cluster the keywords, selected the “cluster” option, and used the Pathfinder algorithm to cut the connection lines to ensure the classification rationality of clustering. The results are shown in **Figure 7**, which reflects the research topics in the field of molecular glues since the 21st century. A total of 19 clusters were obtained. The information on each cluster is shown in **Figure 7**.

As can be seen from **Figure 7**, This study identified 19 distinct research clusters within the molecular glues domain, each highlighting a unique aspect of the field: #0 Drug Design, #1 Protein Degradation, #2 Molecular Recognition | Arginine Rich Peptides, #3 Films, #4 Prostate Cancer | Molecular Glues, #5 Liposomes, #6 TiO2 Nanotubes, #7 Sulfonamide Anticancer Agent, #8 Tissue Adhesives | Surgical Adhesive, #9 Cereblon, #10 Hydrogel, #11 Retinoblastoma, #12 Synthesis, #13 SCC1 | COP9-Initiation Factor Domain, #14 Organic-Inorganic Hybrid Materials, #15 Self-Assembled Monolayers, #16 Biomaterial, #17 Scanning Tunneling Microscopy, and #18 Nuclear Membrane Protein. Different clusters cover different keywords and topics. Clusters # 0,#4,# 7, and # 9 were the hot spots of molecular glue in recent years, which mainly focused on tumor drug development and design. The keywords of clusters #3, #6, and #12 had the earliest average cluster time, mainly on the research in the field of materials science and chemical engineering science, most of which were physical and chemical properties and characterization of molecular glue, and few biological concepts have been involved. Cluster # 2,# 5, # 8, # 10, #16, and # 9 were inspired by the principle of molecular glue action in materials science. The novel concept of molecular glues is gradually attracting attention and maturing in the field of biomedical sciences. What clusters # 1, #11, #13, and #18 discussed were the activity mechanisms and action principles of molecular glue substances at the molecular and cell level in the organism.

**Figure 8:**
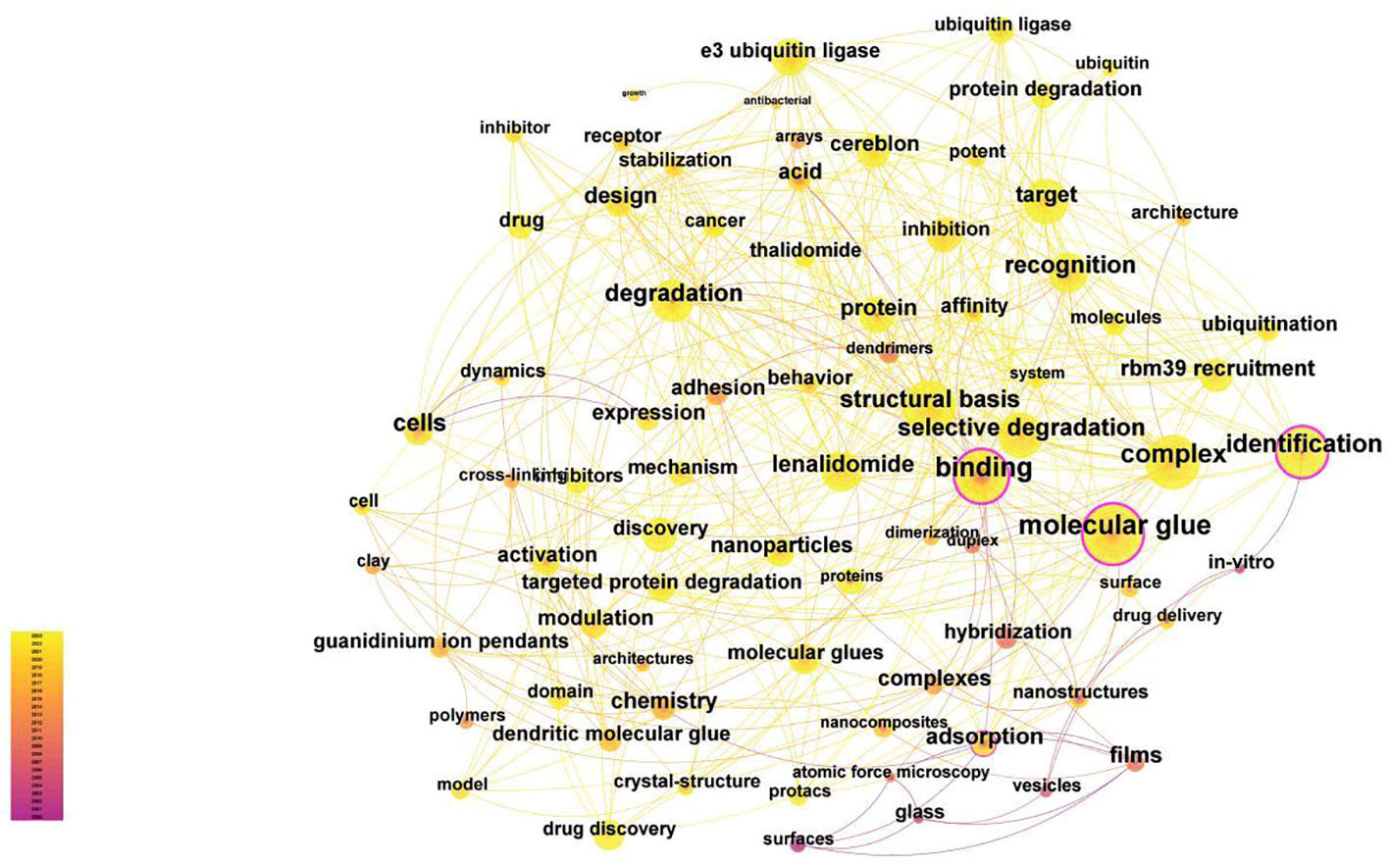
Keywords co-citation analysis map. Using “Keyword” in CiteSpace as the analysis object. A keywords co-citation analysis map with a density of 0.0138 with 523 network nodes and 1887 connections was obtained.

The average year represents the average time for keywords to be researched within the cluster, which also means whether the topic is close to the forefront. For example, the average year of clustering # 11 was 2000, which showed that the research topic of clustering # 11 is the most traditional. The average year of clustering # 0 and #4 was 2019, which meant that the research topics of clustering # 0 and 4 were relatively close to the current research trend. By using “Keyword” in CiteSpace as the analysis object, a keywords co-citation analysis map with a density of 0.0138 with 523 network nodes and 1887 connections was obtained (**Figure 8**). As shown in **Figure 8**, the nodes in the map are relatively dense, and most of the data are connected, with a total connection of 1887.

The five most common keywords were” molecular glue”, “complex”, “binding,” “structural basis,” and “identification.” The keywords with strong intermediary centrality (>0.1) were “molecular glue”, “binding” and “identification”, showing that they were the center of research, having many research directions, and were studied in conjunction with a large number of other keywords.

#### 3.3.2 Analysis of research frontiers

**Figure 9:**
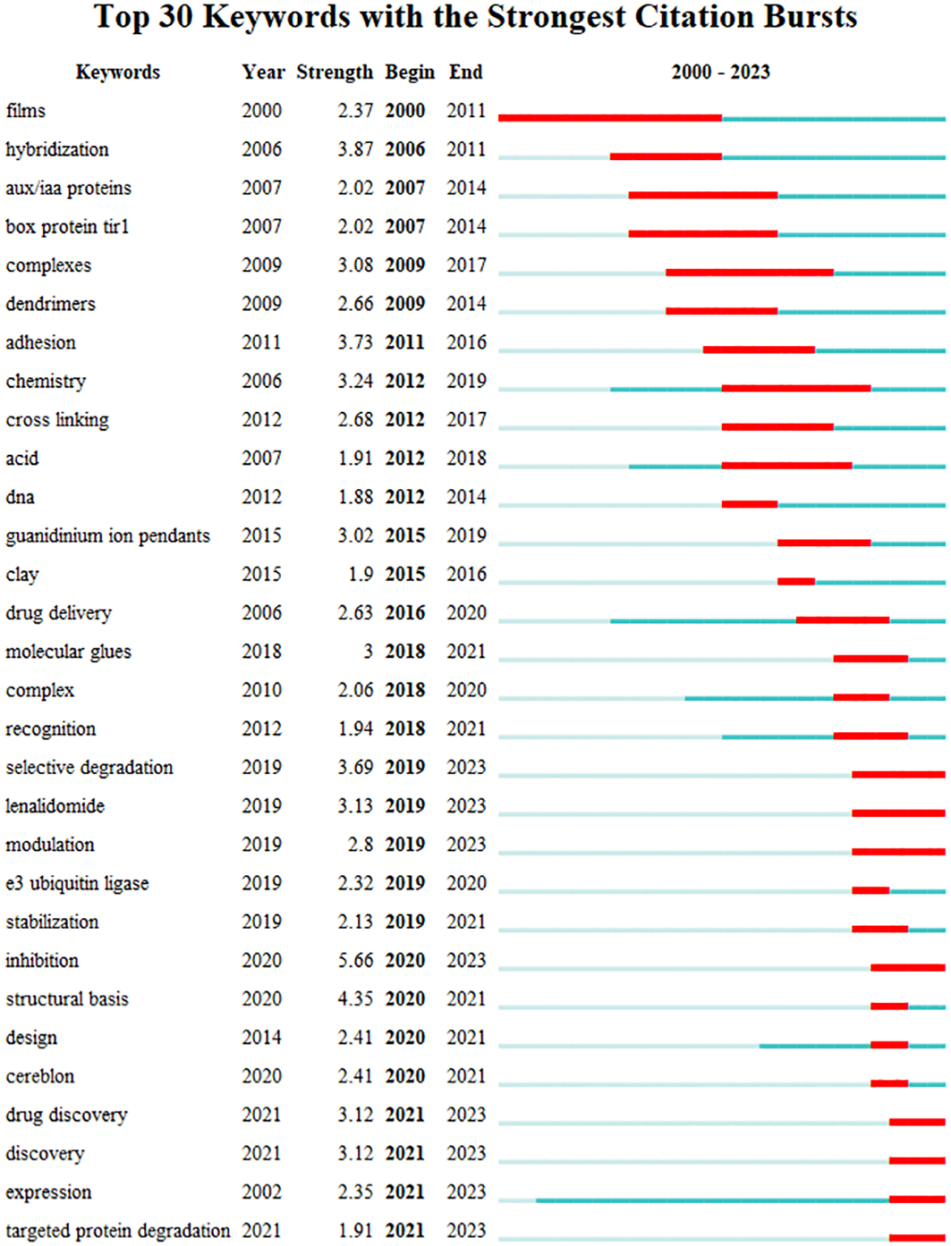
Top 30 keywords with the strongest citation bursts. The Bursts detection algorithm of Citespace software was used to obtain the hot spot evolution map in the field of Web of Science, that is, keyword burst. This study generated the top 30 burst keywords with their burst intensity and duration in the field. The timespan is indicated by a blue line. The time period during when an outbreak appeared is displayed as a red segment, indicating the start and end years of the outbreak duration.”Year” in the figure represents the year when the keyword first appeared. “Strength” indicates the intensity of the keyword outbreak.

In this study, the Bursts detection algorithm of Citespace software was used to obtain the hot spot evolution map of keywords in the field of molecular glue research on the Web of Science, that is, keyword burst. As shown in **Figure 9**, this study generated the top 30 keywords in the field of molecular glue, and the burst intensity and duration were shown in **Figure 9**. A blue line indicates the timespan. The time period during the outbreak in a topic is displayed as a red segment, indicating the start and end years of the outbreak duration.

It can be seen that according to the start time of the study, the studies can be divided into two parts: before 2011 and after 2011. The former had fewer research frontiers and a longer duration of 7.9 years. In the second half of the study, the number of research frontiers was large, and the eruption time was much shorter, which was 2.7 years. This proved that scholars were more active in the second half of the research, and the field developed more rapidly. It can be seen that early studies mainly focused on materials science and chemical engineering science, such as “films,” “hybridization,” “complexes,” and other keywords. Molecular glue, formally defined in the biomedical field, has been studied since 2007. As proven by research on the mechanism of molecular glue, such as “aux/IAA proteins” and “box protein tirl” began to explode. In the later period, the biological mechanism of molecular glue, such as “e3 ubiquitin ligase”, “targeted protein degradation, “and “selective degradation,” began to receive attention. The special properties of molecular glue have made it receive sufficient attention in drug delivery, such as the emergence of keywords like “drug delivery.” In addition, at present, the hottest research has been the development of molecular glue-targeted drugs, such as “drug discovery,” “design,” “lenalidomide,” “cereblon,” and other keywords. The frontiers of research that continue to the present day are drug discovery, discovery, expression, targeted protein degradation, inhibition, modulation, lenalidomide, and selective degradation.

#### 3.3.3 Trends analysis

**Figure 10:**
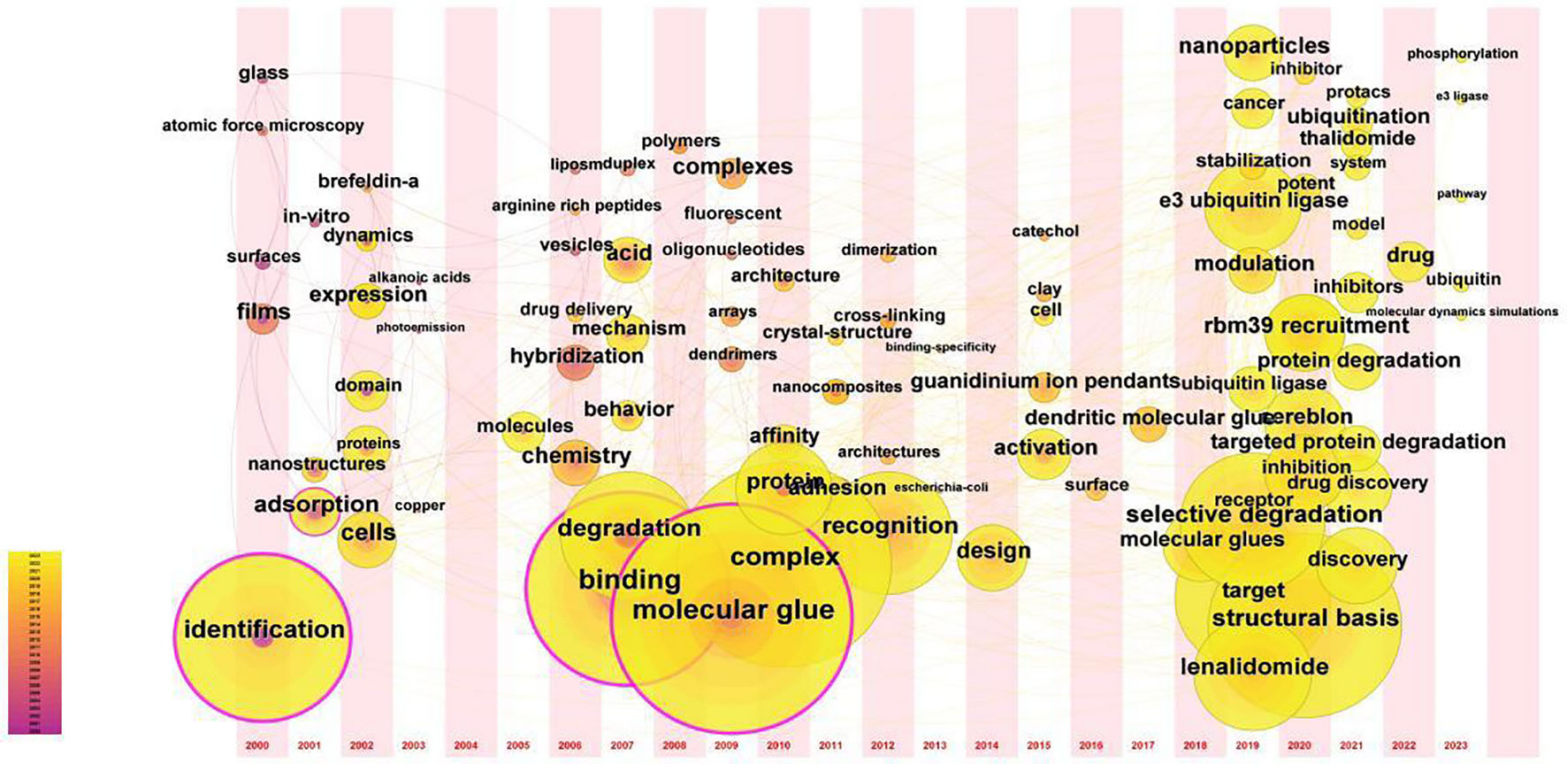
The timezone chart of keywords in regions related to Molecular Glue. The timezone map of the domain was created by analyzing the changes of keywords over time. To generate this map, ‘Timezone’ was selected as the analysis node through Citespace. Specific indicators and thresholds were set as follows: the time slice was set to ‘1’, keywords with smaller nodes were hidden, and the current graph was generated.

We can clearly see that the development of this field could be divided into three stages: 2000-2004, 2005-2015, and 2016-2023(**Figure 10**). In the first stage, through the keywords “films,” “nanostructures,” “*in-vitro*,” and “surface.” It could be seen that the understanding and research of molecular glue in this stage mainly focused on the field of material science and chemical engineering science. In the second stage, Research on molecular glues has progressively gained prevalence in the field of biomedicine and was mainly used in the field of drug delivery, such as “vesicles,” “drug delivery,” “dendrimers,” and “liposomes.” Besides, from the keywords of “degradation,” “arginine-rich peptides,” “duplex,” “protein,” and so on, it can be seen that the biological mechanism of molecular glue began to be studied in the second stage. In the third stage, the design and development of targeted therapeutic drugs of molecular glue began to become very hot, as evidenced by keywords such as “lenalidomide,” “target,” “drug discovery,” “cereblon,” “thalidomide,” “drug,” “cancer,” “rbm39” and so on. In addition, in the present stage, the biological principle and mechanism of molecular glue have also begun to be thoroughly investigated, such as “e3 ligas”, “phosphorylation,” “ubiquitination,” “e3 ubiquitin ligase”, “targeted protein degradation,” “selective degradation” and other keywords.

## 4 Discussion

Our literature trend analysis reveals a marked escalation in molecular glue research since the onset of the 21st century, with an annual average of 16 publications. Prior to 2018, the output was relatively modest, averaging seven articles annually. However, a notable surge in publication volume occurred post-2018, with an average of approximately 43 articles per year, culminating in an average of 60 articles annually over the last three years. Concurrently, the citation count has surged from fewer than five to 1,986, reflecting the growing impact and significance of molecular glue research within the scientific community. This trend underscores an increasing scholarly interest and investment in molecular glue research, indicative of its evolving prominence and potential within the field.

The national analysis reveals a critical role for select countries within the molecular glue research domain, with the United States leading in publication volume (122 articles), evidencing its substantial contributions and leadership. Germany’s prominent intermediary centrality underscores its pivotal role in fostering international collaborations. Close behind, China’s significant publication count signals its ascending influence in the field. This analysis accentuates the predominance of developed countries, especially in Europe and America, in research volume and international cooperation, indicating a concentration of expertise and resources in these areas.

Institutional contributions are led by the Chinese Academy of Sciences, Harvard University, and the University of California system, underscoring their fundamental roles in advancing molecular glue research. Conversely, the University of Tokyo, despite a high publication output, exhibits limited institutional collaboration, reflecting diverse strategies in research partnerships across institutions. This divergence indicates a spectrum of collaboration models, ranging from insular research efforts to extensive international engagements.

Additionally, the author cooperation analysis highlights Takuzo Aida, Luc Brunsveld, and Christian Ottmann as pivotal figures in molecular glue research, with substantial contributions and collaborations. Journal co-citation analysis underscores Nature, Journal of the American Chemical Society, and Angewandte Chemie-International Edition as leading journals, reflecting their central role in publishing influential research in this domain.

We utilized Citespace for keyword clustering revealed 19 distinct clusters, indicating the broad spectrum of research topics within the molecular glue field. Further analysis of the keyword timezone chart reveals a clear division of the development of molecular glue research into three distinct stages. Initially, the focus was concentrated on applications within materials science and chemical engineering science, particularly concerning surface characteristics and structural manipulation at the nanoscale. These explorations set the stage for a subsequent shift towards biomedical research. In the subsequent phase, the concept of molecular glue transitioned into the realm of biomedical research, especially within drug delivery systems. A notable contribution to this was the 2007 study by Professor Ning Zheng’s team, which clarified the role of molecular glues in modulating E3 ubiquitin ligases. Research in this period began to probe the complexities of protein degradation and molecular interaction dynamics. The current phase concentrates on the role of molecular glues in the development of targeted therapeutics. Molecular glues are now integral to drug discovery processes, emphasizing the specificity and selectivity of degradation pathways. The application of molecular glue principles in biomedical fields emerged, reflecting a significant shift towards exploring their potential in drug delivery and disease treatment mechanisms at the molecular and cellular level. Additionally, the transition to a rapid development phase post-2011, with shorter durations of research bursts, underscores a dynamic field experiencing swift changes in focus areas.

## 5 Conclusion

Our analysis underscores the dynamic expansion and diversification in molecular glue research, particularly marked by a notable increase in publications and citations since 2018. This trend reflects a growing global engagement and investment in molecular glue technology for therapeutic applications. The transition from an initial focus on materials science to biomedical applications, especially in drug design, targeted protein degradation, and cancer therapy, signifies a critical evolution in the field’s direction. The United States, Germany, and China emerge as leading contributors, showcasing a concentration of expertise and resources in developed nations that drive advancements and foster international collaborations. Insights from our study reveal an intensified exploration of novel molecular glue frameworks and mechanisms, signaling potential breakthroughs in therapeutic strategies. The identification of unique molecular glue compounds that selectively target and degrade pathogenic proteins opens new pathways for drug development. Additionally, merging molecular glue technology with precision medicine paradigms promises targeted therapies with improved efficacy and minimal side effects. This shift not only illustrates the field’s rapid progression but also its pivotal role in advancing contemporary therapeutic approaches through innovative molecular interventions.

## 6 Limitation

Our analysis reveals a notable concentration of molecular glue research within developed nations, specifically in the United States, Germany, and China, which may not comprehensively represent the global research landscape. This concentration implies a potential underrepresentation of insights from lower-income countries or less resourced institutions, which could contribute unique perspectives and innovations to the field. Furthermore, while our study delineates current trends and anticipates future directions, the inherently rapid evolution of molecular glue research constrains our ability to forecast unexpected breakthroughs or shifts in research focus. Consequently, the speculative nature of projecting future research hotspots and applications may not fully encompass the breadth of possibilities that molecular glue research holds, underscoring the limitations of our analysis in capturing the field’s dynamic and unpredictable trajectory.

## Data availability statement

The original contributions presented in the study are included in the article/supplementary material. Further inquiries can be directed to the corresponding author.

## Author contributions

DL: Conceptualization, Formal analysis, Visualization, Writing – original draft, Writing – review & editing. JC: Formal analysis, Methodology, Visualization, Writing – original draft. ZY: Data curation, Validation, Writing – review & editing. KD: Data curation, Supervision, Validation, Writing – review & editing.
